# Involuntary psychiatric admission: how the patients are detected and the general practitioners’ expectations for hospitalization. An interview-based study

**DOI:** 10.1186/s13033-016-0048-8

**Published:** 2016-03-08

**Authors:** Ketil Røtvold, Rolf Wynn

**Affiliations:** Department of Clinical Medicine, UiT – The Arctic University of Norway, 9037 Tromsø, Norway; Division of Mental Health and Addictions, University Hospital of North Norway, Tromsø, Norway

**Keywords:** Psychiatry, General practitioners, Involuntary admission, Health services, Expectations

## Abstract

**Background:**

In Norway, it is usually GPs that refer patients to involuntary admission. A high proportion of such referrals come from out-of-hours clinics. Little is known about who first initiate the contact between the patients and the referring doctors and which expectations the referring doctors have with respect to the involuntary admissions. The aim of the study was to examine who first detected the patients who were subsequently involuntarily admitted, and to examine the referring doctors’ expectations for the admissions.

**Methods:**

Semi-structured interviews with 74 doctors that had referred patients for involuntary admission at a psychiatric hospital.

**Results:**

Patients who were involuntarily admitted were detected by other branches of the health service (52 %, n = 39), family (25 %, n = 19), and the police (17 %, n = 13). The doctors mentioned these expectations for the admission (more than one expectation could be given): start treatment with neuroleptics: 58 % (n = 43), take care of the patient: 45 % (n = 34), extensive changes to the treatment regime: 37 % (n = 28), solve an acute situation: 35 % (n = 26), and clarify the diagnosis: 22 % (n = 17). Female doctors significantly more often expected that the patients would be examined and treated, while the male doctors significantly more often expected that the patients would be cared for.

**Conclusions:**

Involuntary admissions are typically complex processes involving different people and services and patients with various needs. More knowledge about the events preceding hospitalization is needed in order to develop alternatives to involuntary admissions.

**Electronic supplementary material:**

The online version of this article (doi:10.1186/s13033-016-0048-8) contains supplementary material, which is available to authorized users.

## Background

The involuntary admission of a psychiatric patient will often entail numerous evaluations and actions on several levels [1, 2]. While more is known about characteristics of patients that have been involuntarily admitted to hospitals [3–8], less is known about the often-complex processes leading up to the admission [9–14]. We believe that focusing on the events preceding hospitalization will reveal useful information and enhance our understanding of the dynamics of involuntary admission.

In Norway, the process of involuntary admission will typically start with someone close to the patient, who sees that the patient is in need of help, and makes the decision to contact the patient’s GP or an out-of-hours clinic [15]. In some cases, for instance if there is a public disturbance, it might be the police who sees that the patient is in need of help and brings the patient in contact with the primary health care system [13, 16]. In other cases, it can be the patient’s family that sees that the patient is in need of help and that contacts the primary health care system if the patient himself or herself does not see the need for help. While some have studied which agencies that have been involved prior to psychiatric admissions [17, 18], as far as we know, there have not been any studies that have examined specifically who detected the patients’ need of help in an acute phase and notified the primary care services of this need. Many seriously ill patients are reclusive as part of their illness and therefore get help at a late stage. It may therefore be of importance to patients, their families, and the health service to know who identifies seriously ill patients and initiates contact with the GPs or the out-of-hours clinics.

When a patient is brought to the attention of the GP, the GP will evaluate whether there is a need and a legal basis for involuntary admission to a psychiatric institution. If the patient is admitted involuntarily, a legal evaluation of the involuntary admission will subsequently be made by a psychiatrist or clinical psychologist at the relevant psychiatric institution [19]. According to the Norwegian Mental Health Care Act [19], several conditions must be met in order to involuntarily commit patients to psychiatric institutions. Importantly, the patient must be judged to suffer from a serious mental disorder and the application of compulsory mental health care must be seen as necessary to prevent the person concerned from either (1) having the prospects of his or her health being restored or significantly improved considerably reduced, or it is highly probable that the condition of the person concerned will significantly deteriorate in the very near future, or (2) constituting an obvious and serious risk to his or her own life and health or those of others on account of his or her mental disorder.

The legislation regarding involuntary admission varies between different European countries. Some countries, including Norway, have a system where the decision to admit involuntarily is taken by health professionals and where judges and other legal professionals become involved when these decisions are monitored or appealed [20, 21]. In some other countries, the decision to admit is primarily made by judges and other legal professionals [20, 21].

While the GPs may refer seriously ill patients to involuntary admissions because they consider that the patients meet the required criteria, little is known about which expectations GPs have when referring this group of patients. In order to better understand why patients are referred to involuntary admission, we need to examine the GPs’ expectations. In one study of mainly involuntary admissions, reinstatement of medication, intensive observation, and risk to self or others, were the main reasons for referral [22]. Studies that have examined GPs’ expectations when referring patients with milder psychiatric illnesses to voluntary treatment have suggested that the initiation of treatment that the GP cannot offer or initiate is one important expectation, while having someone share the burden of care or take over the care, are other expectations [23].

The aims of the present study were to find out who first noticed or detected the patients that were subsequently involuntarily admitted to a psychiatric hospital and to examine which expectations the referring doctors had with regard to the involuntary admissions.

## Methods

### Design

This was an interview-based study. The interviews were semi-structured and drew on a tailor-made questionnaire with altogether 19 items (see Additional file 1). For each question we developed several response options. During the interviews, the doctors were first asked the questions without being given any response options and the answers were categorized according to the response options by the interviewer. Only in those cases when the responses did not clearly match any of the response options were the response options read out to the respondents and the respondents asked which response option that was most appropriate. There were also open response options for those cases where no response option matched. The questionnaire has been described in a prior publication [24]. We asked questions about the doctors’ assessments and considerations with respect to the involuntary admission of psychiatric patients. There were also some questions about the doctors themselves, including about their work-experience, type of workplace, and gender. One of the topics we discussed was who the GPs believed had detected that the patients were in need of help (which might even be someone that the GPs had not communicated directly with). We also discussed which needs the GPs felt the patients had that were not fulfilled during regular consultations. We therefore asked the doctors which expectations they had with regard to the involuntary admission in question. The doctors were asked to think about their latest referral during the interview, but without giving any information that would identify individuals. The duration of the interviews was ca. 10 min. The interviews were performed by the first author, who is a psychiatrist. He had not worked with this patient group for several years prior to the present study.

### Setting

The University Hospital of North Norway is the only psychiatric hospital in Troms and Finnmark, the country’s two northernmost counties. The hospital, which has its main location in the city of Tromsø, has a population base of ca. 250,000. The psychiatric clinic has approximately 100 beds at its main location. A majority of the involuntary admissions are to the three acute psychiatric wards, while a smaller number are admitted involuntarily to the two forensic wards and the psychogeriatric ward. Approximately 500 patients are admitted involuntarily each year.

### Sample

The study took place in 2011. We excluded the holidays in order to avoid a high number of temporary doctors. The first author received a list of the doctors who in the previous week had referred patients to involuntary admission as well as the dates of admission. We did not collect any information about individual patients. The relevant doctors’ offices were contacted by phone with a standard request for an interview with the doctor who had made the referral. The doctors who returned the call were given detailed information about the study and asked to participate in a phone interview. About one-third of the doctors returned the call, of which two doctors declined to participate in the study. We lack information about the doctors that chose not to call back or that did not participate in the study. One interview was not completed and therefore not included. Seventy-four interviews were completed in full. Forty-six (62 %) of the participants were male and 28 (38 %) were female. Thirty-nine (53 %) worked at an out-of-hours clinic and 35 (47 %) worked elsewhere. Twenty-four (32 %) had worked more than 10 years, 11 (15 %) had worked between 5 and 10 years, and 39 (53 %) had worked less than 5 years. Twenty of the respondents (27 %) were the patient’s family doctors and 45 (61 %) had some prior knowledge of the patient. The characteristics of the participants have been described in a prior publication [24]. The doctors who chose to participate were typical of the Norwegian general population of general practitioners in terms of gender and work experience [25].

### Analysis

Almost all the doctors’ responses could be classified in the preformulated response options and very few responses were coded in the open response options. The data concerning who the GPs believed detected the patients and concerning the doctors’ expectations were summarized in tables (with n,  %). Due to the relatively small sample size and some table cells with a resulting small n (Table 2), we subsequently grouped the expectations given by the doctors into two main categories (‘Examining and treating’ and ‘Giving care’) and calculated Chi-square statistics for the variables of doctors’ gender, work place, work experience, family doctor/not family doctor, and prior knowledge of patient/no prior knowledge (Table 3). We used a 5 % significance level.

### Ethics

The Regional Committee for Health Research Ethics approved the study (reference P-REK-Nord-2009/1734). No information that could identify patients was collected. The doctors who chose to participate did so after they had received information about the study.

## Results

An overview of who first detected the patients in question is presented in Table 1. Other branches of the health service, followed by the families of patients and the police were those who most often first detected the patients.

**Table 1:** List of who detected/identified the patients

Who detected/identified the patients in question?	Percentage of respondents who listed each agency (n)^a^
1. Other branches of the health service	52 % (39)
2. Family of patients	25 % (19)
3. The police	17 % (13)
4. Friends of the patient	5 % (4)
5. Others	5 % (4)

^a^74 respondents, of which five chose two options

An overview of the referring GPs’ expectations for the admission and characteristics of the GPs is presented in Table 2. For all the doctors, the most expected action at the hospital was starting treatment with neuroleptics (43, 58 %), followed by taking care of the patient (34, 45 %), extensive changes to the treatment (28, 37 %), solving an acute situation (26, 35 %), and clarifying the diagnosis (17, 22 %).

**Table 2:** Expectations of doctors who referred patients to involuntary admission

	Examining and treating			Giving care	
	Treatment with neuroleptics	Implement extensive changes in treatment and follow-up	Resolve an unclear diagnosis	Take care of the patient	Simply solve an acute situation
Gender					
Men	26 (28 %)	13 (14 %)	10 (11 %)	24 (26 %)	19 (21 %)
Women	17 (30 %)	15 (27 %)	7 (13 %)	10 (18 %)	7 (13 %)
Work place					
Out-of-hours clinic	19 (25 %)	11 (14 %)	11 (14 %)	18 (23 %)	18 (23 %)
Other	25 (34 %)	18 (25 %)	6 (8 %)	16 (22 %)	8 (11 %)
Patient’s family doctor					
Yes	15 (37 %)	10 (24 %)	1 (2 %)	9 (22 %)	6 (15 %)
No	28 (26 %)	18 (17 %)	16 (15 %)	25 (23 %)	20 (19 %)
Prior knowledge of patient					
Yes	30 (34 %)	21 (24 %)	6 (7 %)	18 (20 %)	13 (15 %)
No	13 (22 %)	7 (12 %)	11 (18 %)	16 (27 %)	13 (22 %)
Work experience					
<5 years	21 (28 %)	10 (13 %)	11 (14 %)	20 (26 %)	14 (18 %)
5–10 years	8 (35 %)	5 (22 %)	1 (4 %)	5 (22 %)	4 (17 %)
>10 years	14 (29 %)	13 (27 %)	5 (10 %)	9 (18 %)	8 (16 %)
Total^a^	43 (58 %)	28 (37 %)	17 (22 %)	34 (45 %)	26 (35 %)

^a^The questionnaire allowed for multiple answers without any ranking

When examining the responses according to characteristics of the doctors and their relationships with the patients in question, the biggest difference between the male and the female respondents related to the expectation that extensive changes in treatment and follow-up would be implemented (women 27 %, men 14 %). A higher proportion of the men than the women expected an acute situation to be solved (women 13 %, men 21 %) and the patient being taken care of (women 18 %, men 26 %). With respect to work place, a higher proportion of doctors at the out-of-hours clinic expected an acute situation to be solved (23 % out-of-hours clinic, 11 % other) and a higher proportion of the other doctors expected extensive changes in treatment (25 % other, 14 % out-of-hours clinic). When considering the expectations of the patients’ family doctors, we find that while 16 (15 %) of those who were not the patients’ family doctors expected the diagnosis to be clarified, this was the case with only 1 (2 %) of the family doctors. With respect to the prior knowledge of the patient, a higher proportion of those who lacked such knowledge expected the diagnosis to be clarified (18 vs. 7 %) and a higher proportion of those with prior knowledge expected extensive changes in treatment (24 vs. 12 %). A higher proportion of the more experienced doctors expected extensive changes in treatment (27 vs. 13 %) and a higher proportion of the less experienced doctors expected the patient to be taken care of (26 vs. 18 %).

We grouped the doctors’ expectations into two main categories, ‘Examining and treating’ and ‘Giving care’ (Table 3), and calculated between group differences. The only statistically significant difference was for the variable of gender, where the female doctors significantly more often than the male doctors had the expectation that the patients would be examined and treated during the admission (70 vs. 53 %), while significantly more of the male doctors than the female doctors (47 vs. 30 %) expected that the patient would be given care (Chi-square test, χ^2^ = 3.87, p < 0.05).

**Table 3:** Expectations grouped in the categories ‘Examining and treating’ and ‘Giving care’

	Examining and treating	Giving care	Chi-square statistics
Gender			
Men	49 (53 %)	43 (47 %)	χ^2^ = 3.87, p < 0.05
Women	39 (70 %)	17 (30 %)	
Work place			
Out-of-hours clinic	41 (53 %)	36 (47 %)	χ^2^ = 3.00, ns*
Other	49 (67 %)	24 (33 %)	
Patient’s family doctor			
Yes	26 (63 %)	15 (37 %)	χ^2^ = 0.54, ns*
No	62 (58 %)	45 (42 %)	
Prior knowledge of patient			
Yes	57 (65 %)	31 (35 %)	χ^2^ = 2.54, ns*
No	31 (52 %)	29 (48 %)	
Work experience			
<5 years	42 (55 %)	34 (45 %)	χ^2^ = 1.26, ns*
5–10 years	14 (61 %)	9 (39 %)	
>10 years	32 (65 %)	17 (35 %)	
Total^a^	88 (59 %)	60 (41 %)	

* Significance level = p < 0.05

^a^The questionnaire allowed for multiple answers without any ranking

## Discussion

We may assume that most of the patients that were later involuntarily admitted had not sought help on their own, but were detected by others. Some prior studies have examined which agencies that helped patients before they were admitted to psychiatric hospital, and described different ‘pathways to care’ for patients suffering from serious psychiatric illness [17, 18]. In our study, we have asked the GPs to clarify a related but different matter, namely to identify who they believed detected the patients and started the process that lead to the contact between the GPs and the patients. Our study shows that the GPs believed the processes typically were initiated by someone in close contact to the patients on a regular basis, and more than half (52 %) were initiated by the health service and 25 % by family. Only 17 % of the admissions were initiated by the police, according to the GPs.

The high number of patients detected by the health service is probably a result of the fact that this is a group with serious illness and that the group is responsible for a high proportion of re-admissions to hospitals [26]. Consequently, this is a group of patients that typically is followed up by municipal services, including psychiatric home nurses. Half of all involuntarily admitted patients live alone [3]. This is important to remember when we consider the number of patients that were detected by their families. The finding that the family was involved in 25 % of involuntary admissions is comparable to results from other European studies. For instance, in a study from England, Jancovic et al. found that 77 % of involuntarily admitted patients lived alone, and that most of these did not have contact with their families [27]. Studies from other parts of the world suggest a much higher degree of family involvement, and Bola et al. [28] found that families were involved in 63–70 % of involuntary admissions in South Korea. Some of the difference in the proportions detected by family might be explained by differences in legal framework and cultural ties relating to family. Family involvement in involuntary admission is a difficult matter for patients and their families. Family involvement might have long-term consequences for relationships within the family [29], and the next of kin often has mixed feelings about initiating involuntary admissions [27]. Only 17 % were detected by the police, which in part may be explained by the Norwegian regulations and their application -most patients are involuntarily admitted because of their need for treatment and not because they are seen as a danger to themselves or others [26]. Another explanation may be that Norway has a well developed municipal health service, and this health service is able to fulfill a role that the police have to assume in some other countries.

We chose to group the five types of expectations in two main categories, the category of ‘Examining and treating’ (including the expectations ‘Treatment with neuroleptics’, ‘Implement extensive changes in treatment and follow-up,’ and ‘Resolve an unclear diagnosis’) and the category of ‘Giving care’ (including the expectations ‘Take care of the patient’ and ‘Simply solve an acute situation’). While the referring doctors could have several expectations, we believe this categorization is useful in illustrating that other aspects than the purely medical may be important to the referring GPs when considering the involuntary admission of patients [30].

The present study addresses a gap in knowledge regarding GPs’ expectations for involuntary admissions. When looking at the expectations of the group as a whole, the most expected action at the hospital was treatment with neuroleptics (43, 58 %), which was also found to be the main expectation in a different study [22]. Many involuntarily admitted patients use antipsychotics, often several types in combination [31], and an important reason for hospitalization of patients suffering from schizophrenia is failure to adhere to drug regimens [32]. Patients who refuse medication tend to be more ill and more negative to treatment in general [33], and thus at a higher risk of involuntary admission. Nevertheless, 58 % was a high figure, considering that approximately a quarter of involuntarily admitted patients in Norway suffer from non-psychotic disorders, including substance abuse and more serious personality disorders [34, 35], and are consequently not admitted primarily in order to begin or reinstate therapy with neuroleptics.

In 37 % of all cases of involuntary admission, the referring doctors wished the hospital to make extensive changes in the treatment and follow-up of the patients. Here one must take into consideration the fact that a high number of involuntarily admitted patients are rapidly readmitted to hospital after first being discharged [34]. This creates an impression of inadequate treatment and follow-up, and causes frustration. We do not believe that this is a problem that is easily fixed. It may be difficult to create suitable treatment for these patients due to the severity of the illness, a lack of information, and resistance on the part of the patients [36].

Of the GPs that were interviewed, 22 % (n = 17) stated that they expected help with resolving an unclear diagnosis. This finding may be understood in light of a study that showed that as many as a third of the patients that were involuntarily admitted to another Norwegian psychiatric hospital had never been admitted before, and might therefore be more in need of a thorough diagnostic evaluation [37].

In 45 % (n = 34) of the interviews, doctors wanted the hospital to take care of the patient. This may seem obvious, however, we think this is particularly relevant in those cases where the patient has been involuntarily admitted. Studies show that involuntarily admitted patients often have low social functioning, a poor social network with little support, and tend to live alone [3, 38–40]. Consequently, there are few options for those patients who need someone to keep them safe, take care of them, and provide help beyond what we may define as treatment.

A total of 35 % (n = 26) replied that they wanted to resolve an acute situation when they involuntarily admitted the patient. We believe that such a need may occur in situations where there is a crisis, and the patients are overcome by worry and perhaps aggression. In such a situation, it may be difficult to focus on anything but the patient’s behavior [41].

When examining the responses according to characteristics of the doctors and their relationships with the patients in question, we find some interesting differences. While the female doctors more often expected extensive changes in treatment, the male doctors more often expected the patient to be taken care of. This was also the only statistically significant between group difference (see Table 3). Moreover, there was a non-significant tendency that the less experienced doctors were more likely to expect that the patients should be taken care of, while the more experienced doctors expected that the patients would be examined and treated. This could give the impression that the female doctors as well as the more experienced doctors expected more from the hospital than the male doctors and the inexperienced doctors did.

There was also a non-significant tendency that a higher proportion of doctors at the out-of-hours clinic expected the patients to be given care and a higher proportion of the other doctors expected the patients to be examined and treated, a finding that may reflect the role of the out-of-hours clinic doctors as emergency doctors that are expected to prioritize the acute care of patients. Prior studies have suggested that doctors working at out-of-hours clinics have an increased chance of encountering psychiatric patients that are gravely ill [42, 43].

Family doctors know their patients better than doctors who are not the patients’ family doctors, which was reflected in the finding that almost none of the family doctors expected an unclear diagnosis to be resolved during the hospital stay (Table 2). In line with this thinking, those doctors who lacked prior knowledge of the patients were more prone to expecting an unclear diagnosis to be resolved, and those who had such knowledge were more prone to expecting extensive changes in treatment. Both those doctors who were the patients’ family doctors and those who were not, mentioned the need to take care of the patient or resolve an acute situation. One reason for this may be that the patients who were referred to involuntary admission were so ill and perhaps so anxious and aggressive that it was hard find alternatives to hospitalization, in spite of the fact that the family doctors had better knowledge of their patients and the patients’ resources and networks. Another reason might be that they could not separate the need to take care of the patient or resolve an acute situation from what the doctor perceived as the patient’s other needs. The acute situation and the resulting needs for care could not be separated from the needs that arose from lack of medication.

As early as 1968, Bittner commented that a lack of proper alternatives remained an important reason for why the police used coercion against the mentally ill [44]. Do doctors today face a similar situation? We found that the doctors wanted the hospital to make changes to existing treatments or therapy with neuroleptics and many wanted to use hospitalization to solve an acute situation and/or take care of their patient. Maybe it could be possible to find alternatives to involuntary admission in some of these cases and thereby solve what is perhaps more social needs. The picture is complex, however, as the patients that are involuntarily admitted may have many overlapping needs. The clinical and social consequences of illness are important for understanding the dynamics of involuntary admission, and therefore also relevant for the efforts to limit their numbers. We believe that a continued focus on alternatives to involuntary admission is important if the numbers are to be reduced. While research in this area is limited, we believe an increased availability of other types of mental health services, such as low threshold psychiatric home nursing services and assertive community teams, could result in help being given to many patients before the situation becomes too severe and admission is required. Changes made to the legal framework in other countries have not proven effective in reducing the number of involuntarily admitted patients [45, 46].

### Strengths and limitations

Few prior studies have examined the considerations and expectations of GPs that have referred patients to involuntary admission. The study was based on phone interviews with the referring GPs, allowing the GPs to elaborate on, discuss, and clarify their ideas and thoughts. The interviewer was a psychiatrist. This may have affected the interview in a positive way because he could ask for clarifications based on his knowledge of the subject. At the same, time, we are uncertain whether the doctors adjusted their answers to comply with what they thought were expected of them by a figure of professional authority, rather than providing their own assessments.

We have interviewed 74 doctors, a relatively small number in statistical terms, and we must therefore be cautious about generalizing our findings. With this relatively small sample, the possibility of type II errors should be considered. About a third of the doctors we contacted chose to call back in order to participate in the study. It remains a limitation of our study that we have little knowledge of the group that chose not to participate. However, the participants in our study were representative of Norwegian GPs in terms of gender and work experience.

## Conclusions

A majority of the GPs stated that other branches of the health service had detected that the patients were in need of hospitalization. Most of the GPs expected that therapy with neuroleptics would be initiated during the stay at the hospital. Some, however, replied that they wanted the hospital to take care of the patient or help solve an acute situation. Involuntary admissions are typically complex processes involving different people and services and patients with various needs. We need more knowledge about the events preceding hospitalization in order to develop alternatives to involuntary admissions. We believe there is a need for studies that examine possible alternatives to involuntary admission, including more studies that systematically assess the effect of psychiatric home nursing services and assertive community teams on rates of involuntary admission [47].

## Additional file

10.1186/s13033-016-0048-8 In the Supplemental Material Section the questionnaire/interview guide is presented.
