# Supplementary material for: Involuntary psychiatric admission: how the patients are detected and the general practitioners’ expectations for hospitalization. An interview-based study
Source: Int J Ment Health Syst. 2016 Mar 8;10:20. doi: 10.1186/s13033-016-0048-8 (PMC4782338; doi:10.1186/s13033-016-0048-8)
Supplement: Supplementary file 1 — 10.1186/s13033-016-0048-8 In the Supplemental Material Section the questionnaire/interview guide is presented. [file 13033_2016_48_MOESM1_ESM.doc]

**Additional file 1**

**Veiledning for telefonintervju**

**(Guide for telephone interview)**

**Informasjon som gis til legene**

**(Information to the doctors)**

- Presentere meg selv (Introduce myself)
- Opplyse om at jeg gjør et kort intervju i forbindelse med tvangsinnleggelser ved vårt sykehus (Inform that I am doing a brief inteview related to involuntary admissions at our hospital).
- Undersøkelsen er definert som et forskningsprosjekt men har også et kvalitetssikringsaspekt (The study is defined as a research project but it may also be usefult for quality improvement).
- Opplyse om at intervju tar 10-15 minutter (Inform that the interview takes 10-15 minutes).
- Opplyse om at undersøkelsen er godkjent av etisk komité (Inform that the study has been approved by the Regional Medical Ethics Committee).
- Tenk på forhold omkring siste tvangsinnleggelse når du besvarer spørsmål (Think about the last involuntary admission you were involved in when discussing the questions).
- Opplyse om at pasientene skal holdes anonyme (Inform that all patients are to remain anonymous during the interview – no identifying information shall be given).
- Opplyse om at legene blir anonymisert (Inform that the participating doctors will be kept anonymous).

**Spørreskjema/intervjuguide**

**(Questionnaire/interview guide)**

1. *Hvilken legerolle?(Which role does the doctor have?)*

- Fastlege/kommunelege for pasienten (Family doctor/municipalilty doctor for the patient).
- Kommunelege, men ikke for pasienten (Municipality doctor, but not for the patient).
- Kommunelegevikar (Temporary municipality doctor).
- Legevaktslege, men ansatt som kommunelege (Out-of-hours clinic doctor, but working as municipality doctor).
- Legevakt, men ikke ansatt i kommunelegetjenesten (Out-of-hours clinic doctor, but not employed by the municipality).
- Lege ved annen institusjon (Doctor at another institution).
- Privatpraktiserende (Private practice).
- Annet (Other).

1. *Kjennskap til aktuelle pasient?(Prior knowledge about patient in question?)*

- Tidligere hatt konsultasjon (Has had a consultation with the patient before).
- Diskutert/hørt om pasienten (Discussed/heard about the patient before).
- Ingen kjennskap (No prior knowledge).
- Annet (Other).

1. *Hvilket tilleggskriterium ble brukt? (Which criterion was used?)*

- Behandlingskriteriet (Treatment criterion).
- Farekriteriet (Danger criterion).
- Begge kriterier (Both criteria).
- Ikke spesifisert noen av kriteriene (Not specified any criterion).
- Annet (Other).

1. *Argumenter for bruk av aktuelle kriterium? (Reasons for use of criterion).*

- Hendelser/atferd forut for innleggelse (Events/behaviour prior to admission).
- Hendelser/atferd i konsultasjonen (Events/behaviour during the consultation).
- Tidligere erfaringer (Prior experience).
- Annet (Other).

1. *Kunne en valgt annet kriterium enn det som ble brukt? (Could a different criterion have been chosen?).*

- Ja (Yes).
- Nei (No).
- Vet ikke (Don’t know).
- Annet (Other).

1. *Hvis ”kunne ikke valgt annet kriterium”, hvorfor ikke?(If ’could not have chosen a different criterion’ – why not?).*

- Formelle krav ikke til stede (Formalities not fulfilled).
- Liker ikke kriteriet/stigmatiserende (Don’t like the criterion/believe it is stigmatizing).
- Vanskelig kriterium å bruke/manglende kompetanse til å bruke kriterium (Find it difficult to use criterion/feel a lack of competence in using criteron).
- Annet (Other).

1. Hvis ”kunne valgt annet kriterium”, hvorfor ble ikke dette brukt? (If ’could have chosen a different criterion’ – why wasn’t this chosen?).

- Ikke nødvendig med flere enn ett kriterium (Not necessary with more than one criterion).
- Tenkte ikke på det (Didn’t think about it).
- Vil ikke bruke annet kriterium (Did not want to use another criterion).
- Annet (Other).

1. *Mener en at tilleggskriteriene er enkle å bruke?(Do you think that the criteria are simple to apply?).*

- Behandlingskriteriet er enkelt å bruke (The treatment criterion is simple to apply).
- Farekriteriet er enkelt å bruke (The danger criterion is simple to apply).
- Annet (Other).

1. *Ved bruk av farekriteriet, ble det brukt risikotest eller annen risikovurdering? (If the danger criterion was used, did you use an instrument to assess risk?).*

- HCR 20 (The HCR 20 was used).
- Annen test (Another test was used).
- Ingen test/skjønnsmessig vurdering (No test/clinical judgement only).
- Annet (Other).

1. *Hvor hadde en argumenter eller opplysninger fra som førte til bruk av aktuelle kriterium? (From where did the arguments or information that lead to the use of the criterion come)?*

- Fra politi (From the police).
- Fra annen del av helsevesenet (spesifiser) (From a different part of the health service (please specify)).
- Fra familie (From the patient’s family).
- Fra bekjente (From other people who know the patient).
- Hovedsakelig fra selve konsultasjonen (Mainly from the consultation itself).
- Annet (Other).

1. *Hva skulle eventuelt til for at kriteriet ikke var til stede/oppfylt? (Which factors should have been in place in order for the criterion not to be met?).*

- Mulighet for å kunne følge opp pasienten tett kommende dager (The possibility to follow the patient up closely the next days).
- Pasient som ville samarbeide om for eksempel medisinering (That the patient would cooperate, for instance about medication).
- Bedre boforhold (Improved living conditions).
- Mulighet for tettere oppfølging av helsepersonell i pasientens hjem (That the health service could closely follow up the patient in his/her home).
- Familie som kunne hjelpe til (Family that could help).
- Annet (Other).

1. *Var andre momenter enn tilleggskriteriene avgjørende for innleggelsen?(Were there any other factors than the criteria that were of importance to the admission?).*

- Ja (Yes).
- Nei (No).
- *Hvis ja hvilke momenter: (If yes – which factors):*
  - Fastlåst situasjon (Difficult to resolve situation).
  - Manglende tid i konsultasjonen (Not enough available time during the consultation).
  - Manglende ressurser pga. kveld/helg (A lack of resources because of night-time or weekend).
  - Annet (Other).

1. *Ble en rådet til eller fikk instruksjon om bruken av tvang eller ikke? (Were you given any advice or instructed to admit the patient involuntarily or not?).*

- Fra institusjon (From an institution).
- Fra politi (From the police).
- Fra andre deler av helsevesenet (From other parts of the health service).
- Fra familie eller venner (From family or friends).
- Ikke noe råd eller press (No advice or pressure).
- Annet (Other).

1. *Hvem ”fanget opp” pasienten? (Who saw that the patient was in need of help?).*

- Politi (The police).
- Helsevesenet (The health service).
- Sosialetaten (The social services).
- Familie (Family).
- Venner (Friends).
- Andre (Other people).
- Annet (Other).

1. *Opplevde en press fra andre instanser om innleggelse? (Did you experience pressure from anyone to admit the patient?).*

- Politi (The police).
- Annen del av helsevesenet (Another part of the health service).
- Familie (Family).
- Venner (Friends).
- Andre (Other people).
- Ikke press fra noen (No pressure from anyone).
- Annet (Other).

1. *Hvilke forventninger hadde en til behandling ved institusjon? (Which expectations did you have regarding the outcomes of the admission?).*

- Iverksette medisinsk behandling (That medical treatment should be started).
- Ta vare på pasienten (That the patient shold be cared for).
- Kun løse en akutt situasjon (Only to solve an acute situation).
- Utrede uklart sykdomsbilde (To have a patient with an unclear diagnosis examined and diagnosed).
- Iverksette omfattende tiltak for endring av oppfølging eller behandling (To initiate comprehensive measures relating to the care or the treatment of the patient).
- Annet (Other).

1. *Hvor lenge har du arbeidet som lege? (For how long have you worked as a doctor)?*

- *0-5 år (0-5 years).*
- *5-10 år (5-10 years).*
- *Mer enn 10 år (More than 10 years).*

1. *Legens kjønn? (What is your sex?)*

- Kvinne (Female).
- Mann (Male).

*19) Synspunkter og erfaringer ut over spørsmålene eller svaralternativene (Do you have any other comments or experiences relating to the topics that you would like to talk about?).*
